# Corrigendum to “Effect of* Glycyrrhiza* on the Diuretic Function of* Euphorbia kansui*: An Ascites Mouse Model”

**DOI:** 10.1155/2018/5171687

**Published:** 2018-12-16

**Authors:** Ya Lin, Yanqiong Zhang, Erxin Shang, Wenfang Lai, Hongwei Zhu, Yuhua Fang, Qingxia Qin, Haiyu Zhao, Na Lin

**Affiliations:** ^1^College of Pharmacy, Fujian University of Traditional Chinese Medicine, Fuzhou 350122, China; ^2^Institute of Chinese Materia Medica, China Academy of Chinese Medical Sciences, Beijing 100700, China; ^3^Jiangsu Provincial Key Laboratory for Formulae Research, Nanjing University of Traditional Chinese Medicine, Nanjing 210046, China; ^4^Wangjing Hospital, China Academy of Chinese Medical Sciences, Beijing 100102, China

In the article titled “Effect of* Glycyrrhiza* on the Diuretic Function of* Euphorbia kansui*: An Ascites Mouse Model” [1], there were two errors with the parameters in the regression equations. Therefore, the text reading “Ascites volume was defined by the stepwise equation, *Y*_1_ = 6.331*∗X*_1_^2^*∗X*_2_ − 4.16*∗X*_1_^2^ − 0.1637/*X*_2_^2^ + 10.94 (*r* = 0.9845, *P* = 0.0091), displayed in Figure 3(a). Ascites volume/body weight of mice was defined as a dependent variable *Y*_2_ using the multivariate stepwise regression equation *Y*_2_ = 0.06416*∗X*_1_*∗X*_2_^2^ − 0.006046/*X*_2_^2^ + 0.3062 (*r* = 0.9479, *P* = 0.0103) as shown in Figure 4(a).” should be corrected to

“Ascites volume was defined by the stepwise equation, *Y*_1_ = 6.331*∗X*_1_^2^*∗X*_2_ − 4.16*∗X*_1_^2^ − 0.1637/*X*_2_^2^ + 11.62 (*r* = 0.9845, *P* = 0.0091), displayed in Figure 3(a). Ascites volume/body weight of mice was defined as a dependent variable *Y*_2_ using the multivariate stepwise regression equation *Y*_2_ = 0.06416*∗X*_1_*∗X*_2_^2^ − 0.006046/*X*_2_^2^ + 0.4288 (*r* = 0.9479, *P* = 0.0103) as shown in Figure 4(a).”

In addition, the photograph of the renal pathological changes in the Furosemide group in Figure 7(b)-Furo was misplaced. The corrected version of the figure with its description is shown below:

## Figures and Tables

**Figure 7:**
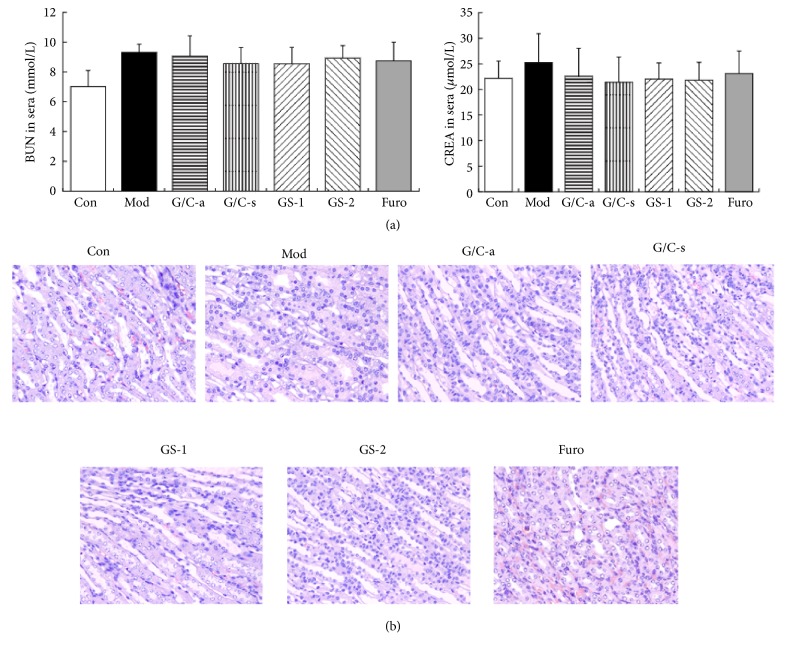
Renal toxicity in H22 HCC ascites mice. (a) Serum levels of BUN and CREA were not altered significantly in any treatment groups. (b) No specific pathological symptoms were detected in different groups. Most kidney cells retained normal structure, without any degeneration or necrosis and edema or swelling of glomerulus and renal tubules. Hematoxylin-eosin (H&E) staining, 400× magnification.
